# Advances in the treatment of newly diagnosed glioblastoma

**DOI:** 10.1186/s12916-015-0536-8

**Published:** 2015-12-08

**Authors:** Brett J. Theeler, Mark R. Gilbert

**Affiliations:** Department of Neurology and John P. Murtha Cancer Center, Walter Reed National Military Medical Center, 8901 Wisconsin Avenue, Building 19, Bethesda, MD 20889 USA; National Institutes of Health, 9030 Old Georgetown Road, Bethesda, MD 20892 USA

**Keywords:** Glioblastoma, High grade glioma, Immunotherapy, Checkpoint inhibitors, Pseudoprogression

## Abstract

Glioblastoma is a refractory malignancy with limited treatment options at tumor recurrence. Only a small proportion of patients survive 2 years or longer with the current standard of care. Gene expression profiling can segregate newly diagnosed patients into groups with different prognoses, and these biomarkers are being incorporated into a new generation of personalized clinical trials. Using the experience from recently completed large scale, multi-faceted, randomized glioblastoma clinical trials, a new clinical trial paradigm is being established to move promising therapies forward into the newly diagnosed treatment setting. Upcoming trials using the immune check-point inhibitors are an example of this changing paradigm and these and other immunotherapies have potential as promising new treatment modalities for newly diagnosed GB patients.

## Introduction

Glioblastoma (GB) is the most common primary brain malignancy in adults and accounts for 45.6 % of all primary brain malignancies. GB has an incidence rate of 3.19 per 100,000 and occurs at a median age of 64.0 years, although GB can occur at all ages [1]. The majority of GBs occur in the cerebral hemispheres, although brainstem, cerebellar, and spinal cord GBs rarely occur [2]. The average overall survival (OS) of GB patients from population series is between 8 and 14 months [3]. Prognosis of GB can be stratified by clinical features, with age younger than 50 years, Karnofsky performance status (KPS) of 70 or better, non-eloquent tumor location, and maximal extent of resection all associated with improved patient outcomes [4, 5].

The backbone of upfront treatment of GB is maximal surgical resection and adjuvant radiotherapy to a dose of 6,000 cGy. Upfront treatment often included nitrosoureas [6], although when added to surgical resection and radiotherapy modest benefit could only be demonstrated by meta-analysis [7]. Temozolomide, an oral alkylating agent with good blood-brain barrier penetration, was developed in the 1990s and showed benefit in the recurrent setting in GBs and recurrent anaplastic gliomas gaining approval for this indication [8]. The randomized phase III, EORTC-NCIC trial 22981/26981, published in 2005, established the current standard of care, including maximal surgical resection followed by radiotherapy with concurrent and adjuvant temozolomide, and demonstrated improved OS of 14.6 versus 12.1 months and increased proportion of 2-year and 5-year survivorship compared to radiotherapy alone [9, 10].

With approximately 5 % of patients surviving 5 years from diagnosis, additional treatment options for newly diagnosed GB patients are needed [10]. Further improvement of upfront treatment of GB is important as approved treatment options for tumor recurrence, including cytotoxic chemotherapy agents, such as nitrogen mustards, bevacizumab, and tumor-treatment fields, are of limited efficacy [11–13]. For example, approximately 20 % of patients with recurrent GBs treated with lomustine (CCNU) are alive and progression-free 6 months after starting treatment. Bevacizumab, a monoclonal antibody against vascular endothelial growth factor (VEGF), is approved by the US Food and Drug Administration (FDA) for use in recurrent GB based on a relatively high rate of radiographic response and improvement in progression-free survival, but with no evidence to date of a significant improvement in overall survival [14].

### Prognostic biomarkers and gene expression profiling

Prior clinical observations noted that secondary GBs, defined as GBs arising from grade II and III infiltrating gliomas, had improved outcomes compared to primary GBs, or those tumors that arise as GBs *de novo*. Primary and secondary GBs were noted to have different gene mutations and to over-express different extra- and intracellular proteins; for example, epidermal growth factor (EGFR) over-expression is more common in primary GBs [15]. Although, when controlling for age and other patient-related factors, the biologic markers of primary and secondary GBs, including EGFR over-expression, are not independent prognostic biomarkers.

The DNA repair enzyme O^6^-methylguanine methyltransferase (MGMT) repairs O^6^- methylguanine adducts. Hypermethylation of the MGMT gene promoter leads to silencing of MGMT expression. This mechanism can counteract the effects of temozolomide which alkylates the O^6^ position on guanine, resulting in futile activation of the mismatch repair system and ultimately apoptosis [16]. In addition to establishing the standard of care, the EORTC-NCIC trial retrospectively demonstrated that patients with MGMT promoter methylation have an improved prognosis compared to those patients with unmethylated promoter regions [17]. In both the EORTC-NCIC trial and another single-institution retrospective study, newly diagnosed GB patients with MGMT promoter methylation treated with upfront radiotherapy alone had improved survival outcomes, suggesting that MGMT promoter methylation may be prognostic regardless of upfront treatment; however, patients with MGMT promoter methylation treated with temozolomide had the best overall survival outcomes in the EORTC-NCIC trial [9, 18].

Given the antagonistic mechanisms of MGMT-mediated DNA repair and temozolomide, it was hypothesized that higher doses of temozolomide may overcome the DNA repair capacity of MGMT [19]. This hypothesis led to the design of RTOG 0525, a randomized phase III trial comparing dose-intense temozolomide (75–100 milligrams per meter squared taken on days 1 to 21 of a 28-day cycle) versus standard dose temozolomide (150–200 milligrams per meter squared on days 1 to 5 of a 28-day cycle). This trial included prospective tissue collection and stratification of both groups by clinical prognostic factors and MGMT promoter methylation. No benefit of dose-intense temozolomide was seen overall, or in the subgroups of MGMT hypermethylated or unmethylated patients. RTOG 0525 confirmed the prognostic significance of MGMT promoter methylation [20]. While an improved prognosis for newly diagnosed patients with MGMT promoter methylation treated with the EORTC-NCIC regimen is established, there is not an alternative regimen for newly diagnosed GB patients with unmethylated MGMT promoters otherwise eligible to receive the EORTC-NCIC regimen. Outside of a clinical trial, it is the authors’ opinion that all patients with a KPS of 70 or greater and an age of 65 years or less should receive the EORTC-NCIC regimen regardless of MGMT promoter methylation status.

As indicated above, age is an important prognostic factor [4]. GB patients over the age of 65 years and those patients with a KPS less than or equal to 60 represent a clinically important group of patients that develop significant toxicity, and there is no prospective evidence of significant benefit from the EORTC-NCIC regimen in these patients. In two retrospective studies of newly diagnosed GB patients aged 65 years and older, including 291 and 237 GB patients respectively, patients with a good performance status (KPS 70 or greater) and a maximal tumor resection significantly benefitted from the EORTC-NCIC regimen [21, 22]. The Nordic trial randomized patients aged 60 years and older to standard radiotherapy (60 Gy in 30 fractions), hypofractionated radiotherapy (34 Gy in 10 fractions), and standard adjuvant dosing of temozolomide, and demonstrated that hypofractionated radiotherapy and standard dose temozolomide alone were associated with better outcomes in patients over the age of 70 years compared to standard radiotherapy [23]. In the ANOCEF phase II trial, patients aged 70 years or older and patients with a KPS of less than 70 were treated with temozolomide, 150–200 milligrams per meter squared for 5 days every 28 days until disease progression. The ANOCEF trial demonstrated improved survival outcomes compared to historical outcomes for supportive care alone [24]. In the ANOCEF trial and the retrospective studies of elderly GB patients referenced above, MGMT promoter methylation was associated with superior survival outcomes [21, 22, 24]. The NOA-08 was a randomized phase III trial comparing dose-intense temozolomide versus standard radiotherapy in patients over the age of 65 years and those with a KPS greater than or equal to 60 [25]. This trial demonstrated prospectively the importance of MGMT promoter methylation in elderly and poor performance status patients as those with MGMT promoter methylation showed a statistically significant benefit of temozolomide and those patients with unmethylated gene promoters benefited from radiotherapy. Thus, MGMT promoter methylation can identify a group of patients with an improved prognosis and is predictive of overall survival with temozolomide treatment in elderly and poor performance status GB patients. An ongoing international randomized phase III trial, including multiple cooperative groups, randomized GB patients aged 65 years and over to a 3-week course of hypofractionated radiotherapy with concurrent and adjuvant temozolomide versus hypofractionated radiotherapy alone (NCT00482677). Currently, the optimal treatment for elderly GB patients should include consideration of KPS, extent of tumor resection, and presence or absence of MGMT promoter methylation, and ideally should be decided in a multi-disciplinary setting.

Initial efforts in genome-wide sequencing and mutational analysis found genes previously associated with GB and other cancers, such as PTEN, EGFR, P53, and PIK3CA, mutated in GBs [26, 27]. However, there were some surprising findings, particularly the discovery of isocitrate dehydrogenase 1 (IDH1) mutations in 10 of 105 GBs [26]. In a larger study of primary brain tumors, IDH1 mutations were found in the majority of World Health Organization (WHO) grade II and III infiltrating astrocytic and oligodendroglial tumors. IDH1 mutations were found in the majority of secondary GBs, and were nearly absent in primary GBs [28]. IDH1 mutant GBs have unique clinical, radiologic, and molecular characteristics. IDH1 mutant GBs are more likely to be frontally located and to be non-enhancing on contrast MRI studies [29]. Mutant IDH1 protein results in neomorphic enzymatic activity and over-expression of an abnormal cellular metabolite, 2-hydroxyglutarate (2-HG). This metabolite can be detected on MR spectroscopy, and provides promise as a novel imaging biomarker for future interrogation as a means of tracking treatment response and tumor progression [30, 31]. Most importantly, IDH1 mutant GBs have an improved prognosis compared to IDH1 wild-type tumors. IDH1 mutations are present in only 5–10 % of GBs overall, and the average OS for IDH1 mutant GBs is 3 years or longer compared to just over 14–16 months for wild-type tumors [32–34]. In a clinical trial setting it is important to define this patient population within the study cohort given the improved survival. Failure to identify patients, particularly in small clinical trials, could lead to misleading results. The majority of IDH1 mutant high-grade gliomas also have MGMT promoter methylation, and IDH1 mutation is a stronger prognostic biomarker than MGMT promoter methylation [32–35]. Combined analysis of IDH mutation and MGMT promoter methylation may improve prognostication over analysis of either biomarker alone, although the prognostic significance of MGMT promoter methylation may be more significant in IDH1 wild-type tumors [35, 36].

The Cancer Genome Atlas (TCGA) and others have used high-throughput sequencing techniques, such as DNA microarray technology, to evaluate large groups of GBs. These studies have defined three or four different subtypes of glioblastomas defined by their gene expression profiles and gene promoter region methylation signatures [37–39]. The TCGA subclasses include proneural, neural, classical, and mesenchymal, and are named based on the functions of the over-expressed genes in each subclass [38]. Evaluating DNA promoter methylation, specifically CpG island methylation pattern, an analysis of GBs in the TCGA found a distinct subset of GBs with CpG island hypermethylation of gene promoter regions, termed the CpG island methylator phenotype (G-CIMP). Nearly all the G-CIMP tumors have IDH1 mutations and a proneural pattern of gene expression, and have an improved prognosis [39]. This distinct subgroup of GBs, with a G-CIMP phenotype and IDH1 mutation, represent only 5–10 % of GBs in total. By contrast, GBs with a mesenchymal gene expression pattern have inferior survival outcomes with an average OS of 12 months or less and make up a much larger proportion, approximately 30 %, of GB patients.

Studies using a genome-wide approach have identified additional mutations that may be important in understanding tumor biology, refining prognostic groups, and which may eventually guide use of targeted therapeutics. Multiple studies have reported oncogene and tumor suppressor genes frequently mutated in other cancers, including PTEN, TP53, PIK3CA, PIK3R1, NF1, RB1, as well as amplification of the PDGFR1A and EGFR receptor tyrosine kinases [40]. Activation of the PI3K/Akt/mTOR and RAS-MAPK pathways are common alterations in GB, and it is worth noting the lack of success to date using targeted therapeutics modulating these pathways in newly diagnosed and recurrent GB. The majority of secondary glioblastomas have IDH1 and TP53 mutations, and serve to mark their evolution from WHO grade II and III lower grade gliomas which share these mutations [32]. Recently, it was discovered that most IDH1 mutant glioblastomas also have ATRX mutations. IDH1 and ATRX mutations are mutually exclusive from the genetic events that typically occur in primary (or *de novo*) GBs, such as EGFR gene amplification and loss of PTEN function [41]. Increased activation of telomerase reverse transcriptase (TERT) is present in most human cancers and allows for telomere maintenance and avoidance of senescence. TERT is among the most commonly mutated genes in GB and further study of the prognostic significance of TERT mutations is warranted, and TERT may provide a target for therapeutic development [42–44].

Finally, rare and recently reported genetic events have been found in GB, which may lend themselves to treatment with currently available targeted agents. These mutations include point mutations in the BRAF gene, BRAF V600E and V600K. BRAF V600E mutations are present in a small minority, fewer than 2 % of GBs, but identification may be important as vemurafenib or dabrafenib, approved for use in metastatic melanoma, may be potential treatments [45]. A recent report found that over 40 % of BRAF V600E mutated, non-melanoma cancers responded to treatment with vemurafenib, and this included three out of four anaplastic pleomorphic xanthoastrocytomas (a rare primary CNS neoplasm) [46]. Unique gene fusions have been discovered in many cancers to date, and GB is no exception as the recently described FGFR-TACC gene fusion produces a fusion protein with oncogenic activity. While FGFR-TACC fusions occur in only 3 % of GBs, study of FGFR inhibition in these patients warrants consideration [47].

The biologic and prognostic importance of these gene expression studies underscore the opportunity to design clinical trials in molecularly defined groups of newly diagnosed GB patients. In the future, the IDH1 mutant, G-CIMP subtype may be excluded from some clinical trials given their improved prognosis with current standard treatment. Likewise, designing clinical trials targeting a particular oncogene or tumor suppressor gene mutation enriched in a specific subset of newly diagnosed GB patients, for example NF1 mutations in mesenchymal GBs, may allow for more efficient trial designs with a higher likelihood of success. A significant challenge for future trial design lies in the sub-dividing of an already rare tumor into smaller groups.

There is mounting evidence that a subset of IDH1 wild-type WHO grade II and III astrocytic and mixed gliomas have similar gene expression profiles to GBs, and have inferior survival outcomes compared to other tumors of similar WHO grade and histology [34, 48]. One large retrospective study demonstrated that IDH1 wild-type anaplastic astrocytomas had inferior survival outcomes to IDH1 mutant GBs [34]. Whether these lower grade tumors should be treated similar to GBs with the EORTC-NCIC regimen has not been systematically studied. Designing specific clinical trials for these patients or including them in the GB clinical trials is under discussion.

**Fig. 1:**
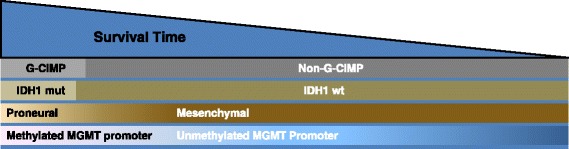
Frequency, overlap, and relative survival of glioblastomas (GBs) (includes GB and all GB variants including gliosarcoma) based on molecular profile. Relative frequency of G-CIMP status, gene expression profiles, IDH1 mutation, and MGMT promoter methylation in GBs. IDH1 mutation and G-CIMP are depicted as discrete categories, while gene expression and MGMT methylation status are depicted as a continuum. GBs with a proneural gene expression profile and IDH1 mutations cluster almost exclusively within G-CIMP GBs, and have improved clinical outcomes (left side). Mesenchymal GBs are exclusively non-G-CIMP and IDH1 wild-type, and have inferior clinical outcomes. G-CIMP, CpG island methylator phenotype; IDH1 mut, isocitrate dehydrogenase 1 gene mutation; IDH1 wt, isocitrate dehydrogenase 1 wild-type gene. Adapted from Theeler BJ et al. [33], with permission from Wolters Kluwer Health, Inc.

GBs can be molecularly stratified at diagnosis into subgroups using IDH1 mutation status, MGMT promoter methylation, and gene expression signature (Fig. 1) [33]. This has prognostic significance and impacts counseling of patients and families on prognosis. Testing for IDH1 mutation by immunohistochemistry, IDH1 and 2 mutation testing by polymerase chain reaction, and commercial testing of MGMT promoter methylation are widely available. Conversely, gene expression profiling is not currently available outside of a research setting. Practically, and at the current time, the treatment of newly diagnosed patients does not change based on the presence or absence of these prognostic biomarkers.

### Changing the current clinical trial paradigm

Since the establishment of the standard of care in 2005, a number of phase II clinical trials were completed in patients with newly diagnosed GB. These trials added various cytotoxic, targeted, biologic, or immunotherapeutic agents to the standard temozolomide regimen. One important lesson from these trials is the necessity of a control arm in order to interpret results [49]. In single-arm, phase II clinical trials in newly diagnosed GB patients, a modest improvement over the EORTC-NCIC trial was noted with various combination therapies, with median OS ranging from 15.1 to 21.2 months (compared to 14.6 months) [49–51]. Patients participating in phase II clinical trials at large institutions may be patients with clinical features resulting in a better prognosis, or perhaps a higher proportion of these patients have favorable molecular features, such as IDH1 mutations and/or MGMT promoter methylation.

Dose-intense temozolomide and bevacizumab have both showed promise in single-arm studies, and demonstrated strong pre-clinical evidence supporting their potential efficacy in newly diagnosed GB [50, 51]. As previously discussed, RTOG 0525 showed no advantage of dose-intense temozolomide, but did show increased treatment-related toxicity in the dose-dense temozolomide arm [20]. The international, phase III CENTRIC study tested the addition of cilengitide, an alphavbeta_3_ and alphavbeta_5_ integrin inhibitor, to the EORTC NCIC regimen in newly diagnosed GB patients with MGMT promoter methylation. The parallel, randomized phase II CORE study tested cilengitide plus standard treatment in newly diagnosed patients with unmethylated MGMT promoters. Despite promising pre-clinical evidence, neither study showed an improvement in OS when adding cilengitide to standard treatment [52, 53]. In a recent development, a treatment device delivering low-intensity, alternating electrical fields, or tumor treatment fields, called the Optune™ system, has gained FDA approval for newly diagnosed glioblastoma based on the results of an interim analysis of the EF-14 randomized phase III clinical trial (http://www.fda.gov/NewsEvents/Newsroom/PressAnnouncements/ucm465744.htm). The publication of this study is awaited to evaluate the place of this treatment modality in newly diagnosed GB patients.

Bevacizumab gained approval for use in recurrent GB in 2009. Despite a lack of definitive evidence that combining bevacizumab with temozolomide enhanced response, and conflicting evidence from single-arm studies combining these agents in the upfront treatment of GB [51, 54], bevacizumab was being incorporated into the upfront treatment of GB at some academic institutions and in the community. In this backdrop, two large randomized, double-blinded phase III clinical trials, RTOG 0825 and AVAglio, randomized newly diagnosed GB patients to either chemoradiation with temozolomide plus bevacizumab or temozolomide plus placebo [55, 56]. Neither trial demonstrated a benefit in OS. AVAglio demonstrated a progression-free survival advantage, while RTOG 0825 did not due to differences in the predetermined statistical model. In the RTOG 0825 study, prospective determination of MGMT promoter methylation status and a 9-gene expression panel (previously shown to be prognostic in GB [57]) were used to stratify patients and for subsequent subgroup analyses. However, no subgroup of patients could be identified that specifically benefited from the combination treatment. RTOG 0825 also demonstrated that patients treated in the bevacizumab arm had increased treatment-related toxicity and inferior scores on symptoms, quality of life, and neurocognitive measures. In a retrospective, subgroup analysis of patients in the AVAglio study, those patients who had tumors which were both IDH1 wild-type and had a proneural pattern of gene expression may have derived survival benefit from the addition of bevacizumab [58]. The proneural subgroup accounts for approximately 25–30 % of newly diagnosed GBs and prospective confirmation of the benefit of upfront bevacizumab in this group of patients is needed. Additional studies of the tumor tissues and patient data collected from these trials are ongoing.

Testing new treatments in patients with recurrent glioblastoma then moving those with an “efficacy signal” to the newly diagnosed setting requires re-evaluation. There have been a paucity of agents tested in the recurrent setting that have been deemed worthy of testing as frontline treatment. Recurrent GB is a refractory disease, and the patients often have a poor performance status and overall health, particularly when compared to newly diagnosed patients. The tumors are often large and unresectable, with significant requirements for corticosteroid treatment to control cerebral edema. Perhaps most importantly, overall the response rate and relative efficacy of therapies in recurrent GB is modest, and it is often difficult to demonstrate efficacy statistically even in agents with significant promise pre-clinically. For example, cediranib, a VEGF-targeted tyrosine kinase inhibitor, had strong pre-clinical, radiographic, and biomarker data to support its use in GB. But cediranib failed to demonstrate benefit over CCNU in a well-designed phase III trial in recurrent GB [11], slowing the development of a potentially promising agent in newly diagnosed GB.

Tumors at recurrence are different from the primary tumor, as treatment with radiotherapy, cytotoxic chemotherapy, contributes to genetic and biologic changes that allow the primary tumor to overcome the host microenvironment and immune system. There is evidence that temozolomide-induced damage to the DNA mismatch repair system results in a hypermutated phenotype with further deficiency in mismatch repair [59]. In one pre-clinical study comparing primary and recurrent tumors, temozolomide-treated WHO grade II astrocytomas had mutations in key intracellular signaling pathways, which were not present in the primary tumor and could mediate treatment resistance to cytotoxic and targeted therapeutic agents [60]. Additionally, some GBs with proneural gene expression undergo a transition to a mesenchymal pattern of gene expression at recurrence, similar to that reported for epithelial cancers [37]. Selecting therapies effective, or ineffective, at tumor recurrence may not predict effectiveness in newly diagnosed patients due to acquired differences between newly diagnosed and recurrent tumors.

### Immune checkpoint inhibitors: a new approach to trial design and treatment of newly diagnosed glioblastoma

The success of immunotherapeutics in previously refractory systemic solid malignancies, particularly metastatic melanoma, has led to significant interest in testing similar therapeutic strategies in glioblastomas. A variety of approaches are currently being tested in GB clinical trials, including peptide- and tumor-based vaccine strategies, oncolytic virotherapy, and adoptive immune strategies, such as autologous infusions of activated T cells [61, 62].

Recurrent GB is a significant challenge for any therapeutic strategy, including immunotherapeutic approaches. Recurrent GB occurs after patients have undergone radiotherapy and multiple cycles of cytotoxic chemotherapy, and in addition corticosteroids are often required to treat cerebral edema resulting in relative immune suppression in many patients. Temozolomide chemotherapy can decrease CD4 T lymphocytes, and patients with reduced CD4 counts have worse clinical outcomes [63]. The relationship between temozolomide-induced lymphopenia and its effect on OS appears to be complex in studies testing vaccination strategies in newly diagnosed glioblastoma patients. In a single-arm study of rindopepimut, temozolomide-induced lymphopenia was associated with improved cellular and humoral immune responses [64], and in another single-arm phase II study testing an autologous formalin-fixed tumor vaccine, patients with grade 3 lymphopenia had improved survival outcomes compared to patients with grade 4 or grade 0–2 lymphopenia [65]. Recurrent GBs may be large and unresectable and pose a significant challenge for any systemic therapy, but may be particularly challenging for immune therapies which need to access an already hostile tumor microenvironment. GBs have decreased expression of major histocompatibility complex (MHC) class I antigen and immune-suppressive proteins, such as IL-10 and transforming growth factor beta, are secreted in the tumor microenvironment [61]. Additionally, the potential risk for immune-related inflammation (pseudoprogression, discussed subsequently) may be more problematic in the recurrent setting.

Dendritic cell (DC) vaccine strategies using autologous tumor lysates or common tumor antigens have been tested in early-phase clinical trials in newly diagnosed glioblastoma patients [66, 67]. This methodology appears to be feasible and safe. Changes in regulatory T cells and CTLA-4 in the systemic circulation correlates with clinical activity, and may provide a means of monitoring therapeutic response [68]. Albeit in uncontrolled, single-arm studies with small numbers of patients, impressive overall median survival rates of 31.4–38.4 months have been observed in newly diagnosed GB patients. Methods to boost the immune responses, such as tetanus toxoid, or with chemokines, such as CCL3, may increase immunogenicity and thereby improve outcomes with DC vaccines in GB patients [69]. Controlled studies, such as the ongoing randomized phase III study using the autologous dendritic cell vaccine, DCVax-L (NCT00045968), are needed to clarify the efficacy of this promising therapeutic strategy.

Gliomas express unique antigens, such as HER-2, TRP-2, gp100, MAGE-1, IL-13 alpha 2, and AIM-2, and the ICT-107, an autologous DC vaccine, has been developed against these antigens [67, 70]. As discussed above, the phase I results of this study were promising (median OS of 38.4 months) [67], and the phase II trial has been completed and the results are forthcoming. Heat shock proteins (HSPs) are expressed during times of cellular and environmental stress, and an autologous HSP-96 peptide complex vaccine has been developed for glioblastoma [70]. A single-arm phase II study has been completed in newly diagnosed GB patients and the results have not yet been published (NCT00905060). In addition to their prognostic and biologic significance, IDH mutations may be a tumor-specific target for immunotherapeutics. In a recent study, an immune response generated against unique epitopes expressed on IDH1 mutated gliomas was successful in a mouse model using a peptide-based vaccine [71].

EGFR variant III (EGFRvIII) is the most common mutation of the EGFR gene in glioblastoma, is present in 25–30 % of GB patients, and is absent in normal tissue [72]. EGFRvIII mutations are associated with poor long-term survival and are mutually exclusive with IDH mutations and G-CIMP gene expression [73, 74]. An EGFRvIII vaccine, called rindopepimut, is a peptide-based vaccination which targets the unique, tumor-specific antigen created by the in-frame deletion of the EGFRvIII gene. Promising results were reported from three phase II trials which added rindopepimut to standard therapy in newly diagnosed GB patients with an EGFRvIII mutation. The median OS was 21.8–23.6 months in these single-arm studies [64, 73, 75]. The randomized, phase III, ACT IV clinical trial testing the addition of rindopepimut to radiotherapy and temozolomide in EGFRvIII mutated, newly diagnosed GB patients has completed enrollment and these results are anxiously awaited.

The FDA approval of the immune checkpoint inhibitors, ipilimumab, pembrolizumab, and nivolumab, in metastatic melanoma has led to significant interest in rapid development of clinical trials in GB. Ipilimumab, a humanized IgG1 monoclonal antibody against cytotoxic T lymphocyte antigen (CTLA-4), has demonstrated durable responses and a significant improvement in OS in metastatic melanoma [76]. Perhaps more importantly as it pertains to GB, it has shown promising activity in patients with melanoma brain metastases without significant central nervous system (CNS) toxicity [77]. The other checkpoint inhibitors, pembrolizumab and nivolumab, are humanized monoclonal antibodies against programmed cell death 1 (PD-1), and are approved for use in metastatic melanoma [78, 79]. Dacarbazine, a cytotoxic chemotherapeutic agent with a similar mechanism to temozolomide, was combined with ipilimumab and combination treatment, and had improved outcomes compared to dacarbazine alone in metastatic melanoma [80]. The sequencing and combination of CTLA-4 and PD-1 blockade is ongoing in clinical trials in melanoma, with results suggesting that combination therapy is more effective but with more treatment-related toxicity [81].

In a study using an immunohistochemical assay, 88 % of newly diagnosed GBs had robust and diffuse expression of PDL-1, the ligand of PD-1. This rate of expression is relatively high compared to other cancers, including melanoma. In this same study, PDL-1 expression was enriched in GBs with mesenchymal gene expression, the subset of GBs which have the worse survival outcomes [82]. In a recent study analyzing PDL-1 expression using both immunohistochemistry and flow-cytometry, PDL-1 expression was reported in 61 % of patients, but the median percentage of cells expressing PDL-1 was 2.77 % with a wide range (0–86.6 %) [83]. Whether expression of PDL-1 on a small sub-population of GB cells will correlate with treatment efficacy will be an important determination in early-phase clinical trials. PDL-1 expression appears to correlate with worsened survival outcomes [83], and the expression of PDL-1 in the majority of GBs provides strong rationale for ongoing clinical trials.

**Fig. 2:**
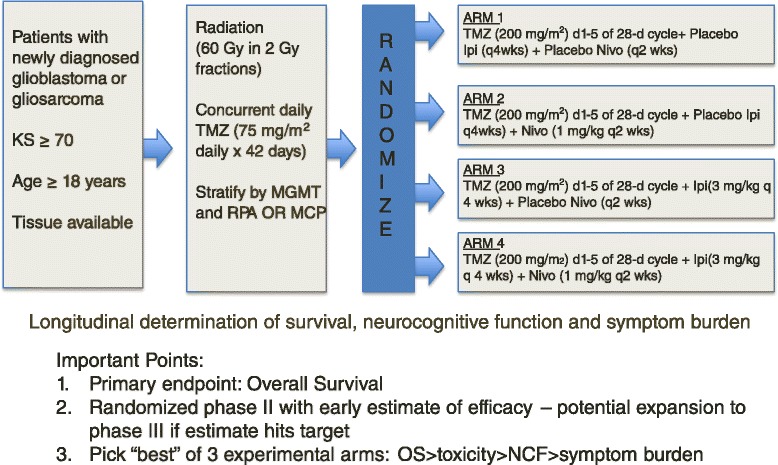
Example of a next generation phase II/III clinical trial for newly diagnosed GB. Patients are randomized after stratification by presence or absence of MGMT promoter methylation (MGMT), clinical factors (recursive partitioning analysis or RPA), and molecular features (gene expression profile or MCP). All patients receive standard temozolomide (TMZ) chemotherapy on days 1 to 42 during radiotherapy and on days 1 to 5 of 28-day cycles during adjuvant treatment. Patients will also receive a combination of placebo, ipilimumab (Ipi), or nivolumab (Nivo) in four treatment arms. A “pick the winner” trial design will be used during phase II to move the most efficacious treatment arm forward into a larger phase III clinical trial. A combination of OS, treatment-related toxicity, neurocognitive function (NCF), and symptom burden will be used to pick the best treatment (arms). Figure was created for this manuscript by the authors

Studies are currently being conducted in recurrent GB using the checkpoint inhibitors, including nivolumab, pembrolizumab, and ipilimumab. The CheckMate 143 trial (NCT02017717) is a randomized phase II trial testing nivolumab alone, nivolumab plus ipilimumab in two different treatment arms versus bevacizumab as an active comparator in recurrent glioblastoma. In another randomized phase II trial, pembrolizumab is being tested alone and in combination with bevacizumab in recurrent glioblastoma (NCT02337491). However, whether a lack of efficacy in recurrent GB or even increased toxicity will be similarly predictive of outcomes in the newly diagnosed setting is questionable, and in our opinion should not preclude or delay development of trials testing checkpoint inhibitors in newly diagnosed patients. A randomized phase II/III trial testing combinations of temozolomide, ipilimumab, nivolumab, and placebo in four different treatment arms to test whether CTLA-4 blockade alone, PD-1 blockade alone, or a combination of both, improve outcomes in addition to standard upfront treatment is currently open and accruing patients (NCT02311920, Fig. 2). This study is designed to take the best experimental arm forward into a fully-powered, phase III, placebo-controlled clinical trial. A phase I/II trial with pembrolizumab and temozolomide in newly diagnosed patients is open and accruing patients (NCT02530502). Additional strategies include adding checkpoint inhibitors to tumor vaccines to increase the immune response; and the upcoming AVeRT trial is a phase I/II study adding nivolumab to the DC vaccine in recurrent high grade gliomas testing this strategy (NCT02529072).

This is not to suggest that upfront trials using checkpoint inhibitors will not be challenging. Systemic toxicity will need to be closely monitored and include autoimmune adverse events, including colitis, endocrinopathies, and dermatologic manifestations [76]; peripheral nervous system toxicity such as Guillain-Barré syndrome and myasthenia gravis have been reported [84]. CNS toxicity, including transverse myelitis, and inflammation of brain parenchyma (in the absence of brain metastasis) have also been reported in the treatment of metastatic melanoma with checkpoint inhibitors [84, 85].

**Fig. 3:**
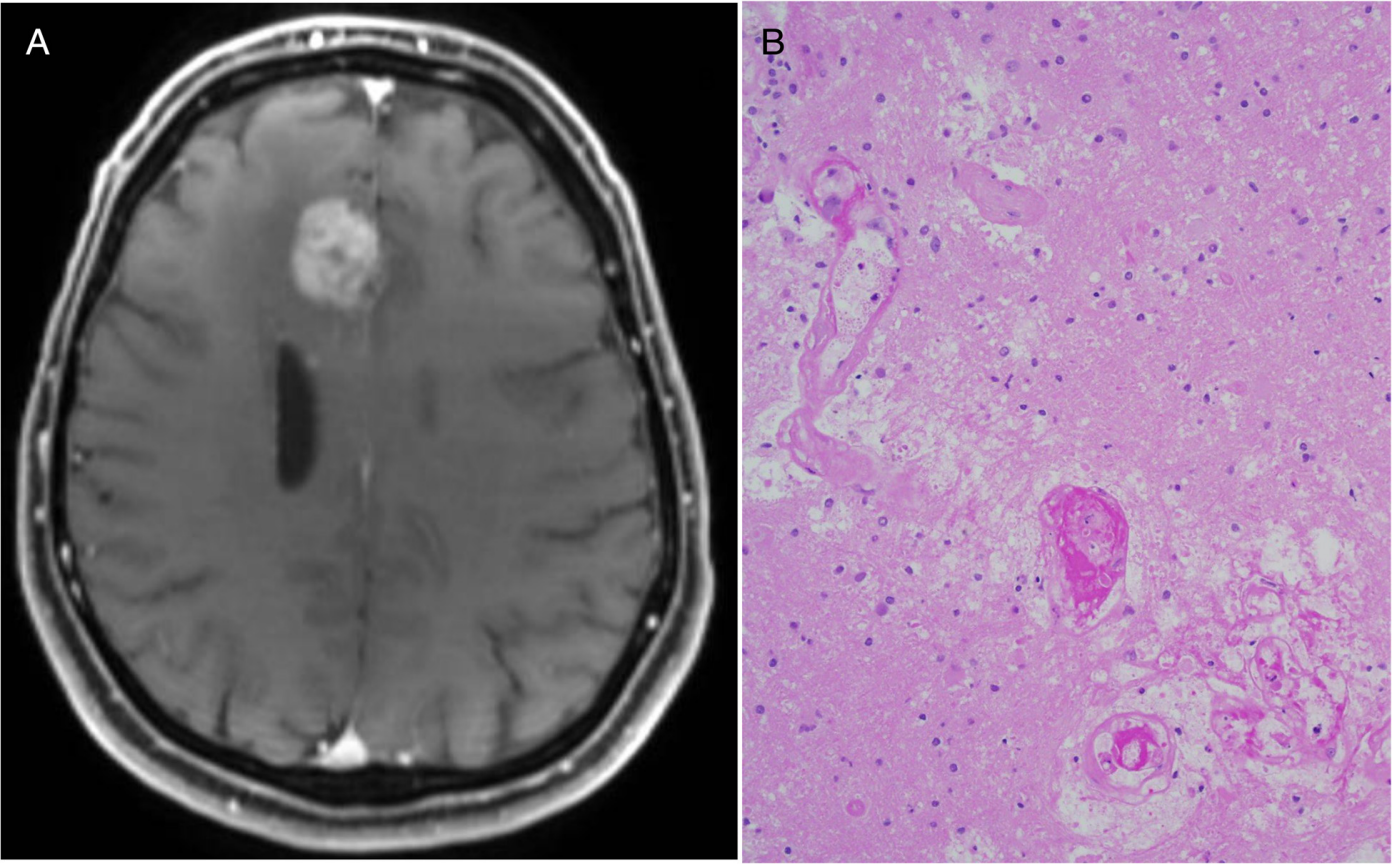
Example of imaging and pathologic features of pseudoprogression in a glioblastoma patient 4 months after completing chemoradiation. **a** Medial, right frontal lobe enhancing mass which was completely resected due to concern for tumor recurrence. Pathology revealed treatment-related necrosis, hyalinized blood vessels, and **b** gliosis (hematoxylin and eosin stain), and a small amount of residual tumor which was not mitotically active (not shown). Figure was created for this manuscript by the authors

Pseudoprogression, operationally defined as reversible radiographic and clinical worsening due to the effects of treatment, typically radiation therapy and temozolomide, is now well recognized. It occurs in 20–30 % of glioblastoma patients after radiation therapy and temozolomide, and usually occurs within 6 months of combined temozolomide and radiotherapy [86]. Pseudoprogression can be difficult to differentiate from tumor progression on standard MRI sequences, advanced MRI sequences, including MR spectroscopy and MR perfusion scans, and even pathologic differentiation can be difficult [87–89]; see Fig. 3 for a typical example of pseudoprogression in a GB patient. Pseudoprogression has been observed in patients with CNS metastasis from metastatic melanoma treated with ipilimumab [90], and in day-to-day practice can make the interpretation of response to radiotherapy and checkpoint inhibitors difficult. When combining checkpoint inhibitors, or other immunotherapeutic agents, with standard upfront therapy, rates of pseudoprogression may be increased, the typical interval during which pseudoprogression occurs may change or be prolonged, and differentiating pseudoprogression from true tumor progression may become an even more vexing problem. Response Assessment in Neuro-Oncology (RANO) guidelines have been developed to help standardize clinical and radiographic assessment in neuro-oncology clinical trials [91]. The RANO effort has been expanded to include immunotherapies, so-called iRANO criteria, to help with the standardization of interpretation of clinical and imaging assessment in immunotherapy trials [92]. Specific immune-related response criteria (iRANO) are needed for numerous reasons as mentioned previously, including an expected prolonged time from therapy initiation to immunologic response, the potential for radiographic worsening initially meeting criteria for progressive disease which may then improve in responders, and the potential for continuing therapy with stable disease or relatively small or otherwise insignificant disease progression in treatment responders [92]. Management of the typical autoimmune complications of checkpoint inhibitors, and determining management strategies for CNS toxicities, will become an important part of ultimately incorporating these therapies into clinical practice.

## Conclusions

Glioblastoma is a refractory malignancy with limited treatment options at tumor recurrence. The standard of care for GB, including maximal resection, radiotherapy, and adjuvant temozolomide is currently recommended for all patients but only a small proportion of patients survive 2 years or longer. IDH1 mutation, MGMT promoter methylation, and gene expression profiling can segregate newly diagnosed glioblastoma patients into groups with different prognoses. While all newly diagnosed GB patients currently receive the same standard treatment, these biomarkers are being incorporated into a new generation of personalized clinical trials. The rapid translation of immune checkpoint inhibitors into the newly diagnosed setting is evidence of a paradigm shift in GB clinical trial design. Checkpoint inhibitors and other immunotherapies are a promising new treatment modality for newly diagnosed GB patients, although carefully designed clinical trials built on the platforms developed for the recently completed large-scale, multi-faceted randomized GB clinical trials will facilitate these efforts. In the case of immunotherapies, special consideration of autoimmune and CNS toxicities will be required to properly evaluate these treatments.
